# Fecal amine metabolite analysis before onset of severe necrotizing enterocolitis in preterm infants: a prospective case–control study

**DOI:** 10.1038/s41598-022-16351-8

**Published:** 2022-07-19

**Authors:** N. Deianova, S. el Manouni el Hassani, E. A. Struijs, E. E. W. Jansen, A. Bakkali, M. A. van de Wiel, W. P. de Boode, C. V. Hulzebos, A. H. van Kaam, B. W. Kramer, E. d’Haens, D. C. Vijlbrief, M. M. van Weissenbruch, W. J. de Jonge, M. A. Benninga, H. J. Niemarkt, N. K. H. de Boer, T. G. J. de Meij

**Affiliations:** 1 Department of Pediatric Gastroenterology, Emma Children’s Hospital, Amsterdam UMC, University of Amsterdam, Vrije Universiteit, location VUmc, (Room PK-1 Z050), Postbox 7057, 1007 MB Amsterdam, The Netherlands; 2 Department of Clinical Chemistry, Amsterdam UMC, Vrije Universiteit, Amsterdam, The Netherlands; 3 Department of Epidemiology and Data Science, Amsterdam Public Health Research Institute, Amsterdam UMC, Vrije Universiteit, Amsterdam, The Netherlands; 4 Department of Neonatology, Amalia Children’s Hospital, Radboud UMC, Radboud Institute for Health Sciences, Nijmegen, The Netherlands; 5 Division of Neonatology, University Medical Center Groningen, Beatrix Children’s Hospital, University of Groningen, Groningen, The Netherlands; 6 Department of Neonatology, Emma Children’s Hospital, Amsterdam UMC, University of Amsterdam, Amsterdam, The Netherlands; 7 Neonatal Intensive Care Unit, Department of Pediatrics, Maastricht UMC, Maastricht, The Netherlands; 8 Neonatal Intensive Care Unit, Amalia Children’s Center, Isala, Zwolle, The Netherlands; 9 Neonatal Intensive Care Unit, UMC Utrecht, Wilhelmina Children’s Hospital, Utrecht, The Netherlands; 10 Amsterdam Gastroenterology Endocrinology Metabolism (AGEM) Research Institute, Amsterdam UMC, University of Amsterdam, Tytgat Institute for Liver and Intestinal Research, Amsterdam, The Netherlands; 11 Department of Pediatric Gastroenterology, Emma Children’s Hospital, Amsterdam UMC, University of Amsterdam, Vrije Universiteit, Amsterdam, The Netherlands; 12 Department of Neonatology, Máxima Medical Center, Veldhoven, The Netherlands; 13 Department of Gastroenterology and Hepatology, Amsterdam UMC, Vrije Universiteit Amsterdam, Amsterdam Gastroenterology Endocrinology Metabolism (AGEM) Research Institute, Amsterdam, The Netherlands

**Keywords:** Metabolomics, Gastrointestinal diseases, Infant necrotizing enterocolitis, Neonatology, Preterm birth, Diagnostic markers

## Abstract

Infants developing necrotizing enterocolitis (NEC) have a different metabolomic profile compared to controls. The potential of specific metabolomics, i.e. amino acids and amino alcohols (AAA), as early diagnostic biomarkers for NEC is largely unexplored. In this multicenter prospective case–control study, longitudinally collected fecal samples from preterm infants (born <30 weeks of gestation) from 1–3 days before diagnosis of severe NEC (Bell’s stage IIIA/IIIB), were analyzed by targeted high-performance liquid chromatography (HPLC). Control samples were collected from gestational and postnatal age-matched infants. Thirty-one NEC cases (15 NEC IIIA;16 NEC IIIB) with 1:1 matched controls were included. Preclinical samples of infants with NEC were characterized by five increased essential amino acids—isoleucine, leucine, methionine, phenylalanine and valine. Lysine and ethanolamine ratios were lower prior to NEC, compared to control samples. A multivariate model was rendered based on isoleucine, lysine, ethanolamine, tryptophan and ornithine, modestly discriminating cases from controls (AUC 0.67; *p* < 0.001). Targeted HPLC pointed to several specific AAA alterations in samples collected 1–3 days before NEC onset, compared to controls. Whether this reflects metabolic alterations and has a role in early biomarker development for NEC, has yet to be elucidated.

## Introduction

Necrotizing enterocolitis (NEC) is a gastrointestinal disease characterized by intestinal necrosis and a high risk of intestinal perforation. It is one of the leading mortality causes in preterm infants, with reported incidence of 5–15% and a mortality rate up to 30%^1,2^. Survivors often suffer life-long health consequences, including intestinal failure, impaired growth and psychomotor development^3,4^.

As infants with NEC can deteriorate within hours, early diagnosis is of critical importance for initiation of treatment^5^. Clinical and radiographic signs, such as emesis, abdominal distention and portal vein gas, are non-sensitive features, especially in infants born at early gestational ages^6^. Predictive biomarkers for NEC are lacking, despite that they are needed to improve neonatal health care and outcome^7–9^.

Fecal metabolomics is an emerging field in the non-invasive biomarker development for various diseases, including NEC^9^. Metabolic profiles are associated with host metabolism and microbiota function, of which alterations potentially reflect pathophysiological processes in NEC^10^. For example, fecal volatile organic compounds (VOC) patterns, have been associated with NEC, already before clinical onset^11–13^. Also liquid chromatography/mass spectrometry (LC–MS) and nuclear magnetic resonance (NMR) metabolic profiling have potential for discriminating infants with NEC from controls^14,15^. A specific metabolomics group of interest is amino acids and amino alcohols (AAA). AAA have various metabolic functions, including growth, gastro-intestinal development and immunogenic functions, processes which can be impaired in NEC^16–19^. As pathophysiological processes are likely to begin before appearance of clinical symptoms, changes in AAA profiles could hypothetically serve as early biomarkers for NEC.

In this proof of concept case–control study, we investigate the potential of targeted fecal AAA analysis as non-invasive biomarkers for NEC in infants with severe stages of NEC (Bell’s stage IIIA/B), which are associated with more unfavorable short and long term outcome, compared to milder stages^20^. This study was embedded in a large national cohort study in which daily fecal samples were collected in preterm neonates to find biomarkers for NEC and sepsis^21^.

## Results

### Baseline characteristics

**Figure 1:**
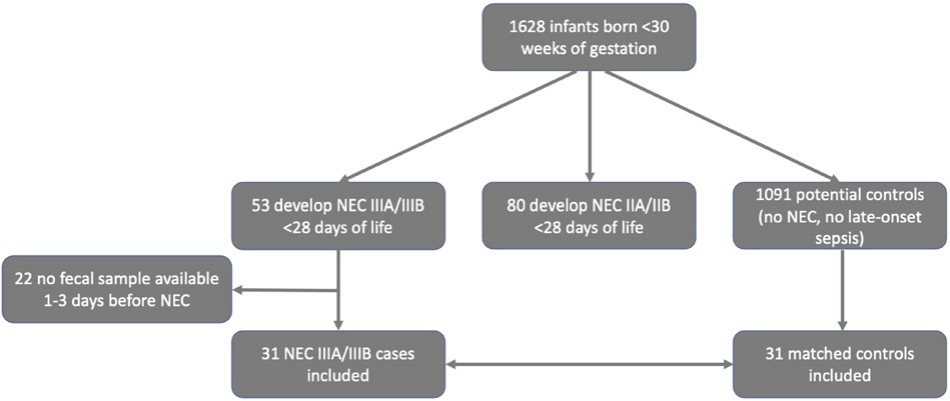
Flow chart of study subject enrollment. *NEC necrotizing enterocolitis*.

In total 1628 infants were included, of whom ultimately 28 were diagnosed with NEC IIIA and 25 with IIIB (Fig. 1). One hundred twenty-two fecal samples were available from 31 cases on 1, 2 and/or 3 days prior clinical onset (i.e. t_-1_ and/or t_-2_ and/or t_-3_, resp.). Baseline characteristics of these NEC cases and 31 gestational age-matched controls are depicted in Table 1.

**Table 1:** Baseline demographic, clinical and sampling characteristics.

	NEC (n = 31)	Controls (n = 31)	p value
**Center of birth (n [%])**			1.00
Centre N^o^ 1–2–3–4–5-6-7-8	8 [26]–6 [19]–1 [3]–3 [10]–2 [7]–4 [13]–1 [3]–6 [19]	8 [26]–6 [19]–1 [3]–2 [7]–2 [7]–3 [10]–2 [7]–7 [23]	
Gestational age *weeks* (median [IQR])	26+5 [25+2–27+6]	27 [26+1–27+6]	0.56
Birth weight *grams* (median [IQR])	960 [755–1150]	988 [840–1150]	0.46
Gender *female* (n [%])	11 [36]	14 [45]	0.44
Singleton (n [%])	21 [68]	21 [68]	1.00
Mode of delivery *vaginal* (n [%])	17 [55]	19 [61]	0.61
Apgar score 1 min (median [IQR])	6 [5–8]	6 [3–7]	0.35
Apgar score 5 min (median [IQR])	8 [6–9]	7 [6–8]	0.14
**Average feeding practice from birth to t**_**-1**_**:**			0.14
Human milk > 75% per day (n [%])	20 [65]	22 [71]	
Formula milk > 75% per day (n [%])	4 [13]	1 [3]	
Combination of Human and Formula milk (n [%])	5 [16]	3 [10]	
Missing values (n [%])	2 [6]	5 [16]	
Primarily enterally fed at t_-1_—*Enteral* *feeding* *percentage* > *75%* *of* *total* *volume* (n [%])	20 [65]	20 [65]	0.77
Missing values (n [%])	5 [16]	4 [13]	
Cumulative antibiotic administration by t_-1_ days (Median [IQR])	6 [3–7]	4 [3–5]	0.17
Invasive ventilation at t_-1_ (n [%])	8 [26]	4 [13]	0.22
**NEC modified Bell’s stage**		NA	NA
IIIA (n [%])	15 [48]		
IIIB (n [%])	16 [52]		
Age at diagnosis (t_0_) *days* (median [IQR])	11 [8–14]	NA	NA
Laparotomy during NEC episode (n [%])	19 [61]	NA	NA
**Late-onset sepsis prior to sampling (n [%])**	12 [39]	NA	NA
Gram-negative LOS (n [%])	2 [6]		
Gram-positive LOS (n [%])	9 [29]		
Candida LOS (n [%])	1 [3]		
**Sample availability (total count)**	59	54	
Time point t-1 (n [% of total samples])	16 [27]	17 [31]	0.85
Time point t-2 (n [% of total samples])	22 [37]	20 [37]	
Time point t-3 (n [% of total samples])	21 [35]	17 [31]	
Sample age *months* (median [IQR])	79 [65–92]	80 [70–99]	0.21

*IQR* interquartile range*,* *n* number of infants, *LOS* late-onset sepsis, *NEC* necrotizing enterocolitis, *t*_*0*_ day of diagnosis, *t*_*-1*_ 1 day prior diagnosis, *t*_*-2*_ 2 days prior diagnosis, *t*_*-3*_ 3 days prior diagnosis*.*

Cases and controls did not differ in terms of baseline demographics, feeding practice, antibiotic use, nor the need for invasive ventilation prior to sampling. Twenty cases (65%) were treated for sepsis in the week prior to NEC. One case had a focal intestinal perforation prior to NEC. Nineteen cases (58%) deceased after NEC diagnosis. Of the control infants, one case was reported with *Staphylococcus* *aureus* early-onset sepsis (EOS) and no infants developed late-onset sepsis (LOS).

### Amino acid analysis

Samples from cases and controls with similar GA (± 4 days) were matched based on postnatal age (± 1 days). AAA analysis was performed by means of high performance liquid chromatography (HPLC). Compounds which could not be quantified in > 50% of fecal samples in at least one of both study groups, were excluded from statistical analysis. Based on 28 remaining AAA, the standard likelihood ratio test revealed seven relative AAA levels significantly altered in infants developing NEC, compared to controls (Table 2). The best fitting logistic regression model consisted of five AAA markers ethanolamine, isoleucine, lysine, ornithine and tryptophan, resulting in an AUC of 0.67, significantly better than random (p < 0.001) (Figure S1).

**Table 2:** Relative amino acid and amino alcohol (AAA) concentrations (% of total AAA concentration) in cases vs. controls on pooled samples from 3 to 1 day(s) before diagnosis.

	NEC	Controls	likelihood ratio test	
	Median [IQR]^a^	Median [IQR]^a^	Raw *p* value	BH-FDR
**Essential amino acids**				
Histidine	2.50 [1.51–3.14]	2.14 [1.69–2.92]	0.35	0.55
Isoleucine^†^*	3.52 [2.44–4.61]	2.10 [1.35–3.25]	0.001	0.03
Leucine*	8.42 [5.80–11.48]	6.45 [3.94–9.30]	0.01	0.06
Lysine^†^*	3.61 [1.53–6.39]	6.12 [2.75–8.23]	0.02	0.07
Methionine*	0.44 [0.13–0.81]	0.19 [0.04–0.57]	0.004	0.04
Phenylalanine*	3.83 [2.82–4.90]	2.85 [1.91–4.18]	0.006	0.04
Threonine	2.93 [1.40–5.20]	2.18 [1.34–4.46]	1.00	1.00
Tryptophane^†^	2.15 [0.80–3.05]	1.14 [0.00–2.07]	0.06	0.19
Valine*	6.22 [4.20–7.78]	4.70 [3.45–6.36]	0.002	0.03
**Conditionally essential amino acids**				
Arginine	0.48 [0.15–2.10]	0.66 [0.23–1.11]	0.42	0.62
Cystine	0.64 [0.11–1.32]	0.82 [0.15–1.36]	0.49	0.65
Glutamine	1.07 [0.10–1.93]	0.82 [0.34–1.87]	0.81	0.87
Glycine	4.71 [3.96–6.68]	5.09 [3.29–7.36]	0.50	0.65
Proline	4.17 [2.60–6.47]	3.69 [2.80–5.13]	0.79	0.87
Serine	0.93 [0.55–2.16]	1.21 [0.77–2.36]	0.57	0.67
Tyrosine	2.89 [0.73–4.48]	1.71 [0.42–3.96]	0.26	0.46
**Non-essential proteogenic amino acids**				
Alanine	8.38 [6.12–10.68]	7.30 [4.71–10.53]	0.31	0.51
Asparagine	0.00 [0.00–0.81]	0.17 [0.00–1.44]	0.23	0.46
Aspartic acid	4.27 [1.41–7.32]	5.02 [2.78–10.11]	0.24	0.46
Glutamic acid	12.44 [6.58–18.90]	14.26 [3.65–21.31]	0.90	0.94
**Non-proteogenic amino acids**				
Apha-aminobutyric acid	0.12 [0.00–0.45]	0.00 [0.00–0.35]	0.53	0.64
Citrulline	1.00 [0.29–1.86]	0.99 [0.26–3.09]	0.51	0.65
Gamma-aminobutyric acid	0.18 [0.00–4.22]	0.00 [0.00–1.33]	0.22	0.46
Ornithine†	0.86 [0.23–2.56]	0.52 [0.19–1.47]	0.11	0.29
Sulfo-l-cysteine	3.66 [0.00–8.44]	9.83 [0.10–17.26]	0.05	0.17
Taurine	0.18 [0.01–0.53]	0.09 [0.00–0.76]	0.12	0.30
**Amino-alcohols**				
Ethanolamine^†^*	1.02 [0.50–1.87]	1.93 [1.18–2.78]	0.01	0.06
Phophoethanolamine	0.35 [0.10–1.10]	0.58 [0.00–1.69]	0.23	0.46

*BH-FDR* Benjamini–Hochberg False Discovery Rate as determined by univariate analysis, *IQR* interquartile range.

^a^Median and IQR of percentage of total amino acids concentration; *Significance level of BH-FDR < 0.1; ^†^Markers of the rendered model by multivariate analysis.

## Discussion

In this prospective case–control study, we investigated preclinical AAA profiles in longitudinally collected fecal samples of infants developing severe NEC (Bell’s stage IIIA and IIIB). During the 3 days preceding clinical onset, seven metabolites were altered in fecal samples, compared to controls. A set of five AAA could moderately predict NEC with an accuracy of 0.67. While, to date, no data are available on the diagnostic value of fecal AAA patterns, several serum and urinary amino acids have previously been proposed as potential NEC biomarkers. Becker et al., e.g., showed reduced levels of serum alanine, glutamine, histidine, lysine, threonine and urea cycle intermediates arginine and ornithine to be associated with NEC, as measured by HPLC on dried blood spots of 45 infants (13 cases vs. 32 controls) on day 7, 14 and/or 21 of life^22^. Together with arginine, decrease in serum citrulline, a third urea cycle intermediate, has been reported in NEC^22–24^. Conversely, multivariate analysis in the current study only pointed to a slight increase of fecal ornithine before NEC diagnosis, and NMR analysis performed by Thomaidou et al*.* showed increased urinary arginine on the day of NEC diagnosis^15^. Although the analyses in blood, urine and feces have been performed in different patients and with different techniques, it would be interesting to explore the role of urea cycle intermediates in NEC pathophysiology in different body compartments.

Thomaidou et al*.* furthermore, reported urinary tryptophan and proline levels to be reduced on the day of NEC diagnosis, as measured by untargeted NMR analysis in 15 cases vs. 15 controls^15^. Inversely, the current multivariate analysis of fecal AAA shows a positive correlation between NEC and fecal tryptophan levels. The potential association of tryptophan disturbance and NEC has previously been suggested by the anti-inflammatory role of its metabolite indole-3-lactic acid (ILA) in the immature intestine^25^. We hypothesized that intestinal loss of tryptophan might be associated with impairment of anti-inflammatory processes.

Inverse correlations of fecal and plasma amino acids have, furthermore, been appreciated in inflammatory bowel diseases (IBD)^26^. This group of conditions are reported to share several pathophysiological mechanisms with NEC, including impaired mucosal defenses, disruption of commensal bacteria, and inappropriate immune responses^27^. In accordance with our findings, increased levels of fecal essential amino acids isoleucine, leucine, valine and phenylalanine have been reported in de novo IBD, compared to controls^26^. Conversely, plasma methionine, leucine, lysine, phenylalanine, tryptophan and valine are reported to be decreased in plasma samples from IBD patients with increased disease activity^28^. Whether potential opposite correlations between fecal AAA and NEC, on the one hand, and systemic AAA and NEC on the other, are a result from impaired enteral absorption or increased intestinal leakage, e.g. due to impaired tight junction function, remains to be explored^29–31^.

A particular strength of this study, compared to available literature, is that fecal samples were longitudinally collected at predefined time points before NEC onset, rather than on fixed days of life, or only after appearance of clinical features^15,22^. This allowed for exploration of the potential of fecal metabolomics as early biomarkers. Secondly, all detailed demographic and clinical data were collected daily in a predefined structured manner, allowing for careful matching of the cases with controls and minimization of intergroup variability. Another strength is the strict selection of infants with severe NEC, Bell’s stage IIIA and IIIB. As NEC has been suggested to be a heterogeneous mix of disease phenotypes, rather than one entity, selecting specific stages potentially offers more metabolic homogeneity and limits intragroup variability^32^. Moreover, by only including infants developing extended disease (stage IIIA and IIIB) in the days following NEC diagnosis, we focused on the population with the highest mortality and prolonged hospital stay, compared to other disease stages^33,34^. This group was prioritized because of its potentially highest need for early diagnostic and treatment measures, compared to milder forms of the disease. Lastly, the analyzing technique allowed for specific determination of 42 AAA, rather than untargeted techniques, such as NMR, the latter which are less sensitive and require more material^35^.

Limitations include the relatively small sample size per time point, which only allowed statistical analysis of pooled samples. As a consequence, a possible stronger effect 1 day before clinical onset could be underestimated by a weak or absent effect 3 days before onset of symptoms.

Secondly, not all known influencing factors on AAA profiles were corrected for by the matching procedure. For example, in line with available literature, concurrent blood stream infections were common in infants developing NEC^36^. As both the disease process and treatment of late-onset sepsis (LOS) could influence AAA profiles, differences between LOS-associated and non-LOS-associated NEC, as well as from NEC-free LOS, should be considered in the future. Additionally, feeding type categories were arbitrarily chosen with a 75% cut-off for human and formula milk, respectively, rather than continuous percentages of human milk intake. However feeding type was similar in both groups, we could have missed subtle differences in AAA related to feeding type between cases and controls^37,38^. Furthermore, sample storage conditions could have an additional influence on AAA levels, as has been reported in, i.a., BCAA’s measured by NMR analysis^39^. Although storage conditions and time were comparable between cases and controls and we, therefore, expect limited storage associated bias in our study, fresh sample analysis should be performed in the future, similar to clinical practice work flow.

Thirdly, only NEC episodes starting within the first 28 days of life were included. Based on previous research, we expect a small proportion of infants, especially those born at an early gestational age, to develop NEC beyond the 28th day of life^40^. As microbiota composition, microbiota/host interactions, and potentially AAA profiles, change with advancing postnatal age, the current results might not be transferrable to older children developing NEC^41^.

Furthermore, by excluding milder NEC cases and limiting the clinical heterogeneity between cases, our findings cannot be transferred to other stages of NEC, which, although to a lesser extent, hold substantial short and long term risks^20^. Finally, a limitation inherent to the observational character of the study is that it merely allows to speculate about potential pathophysiological mechanisms in different body compartments, as there are no simultaneous serum and/or urine samples.

To our judgement, further studies on the potential of AAA in NEC recognition are warranted. The involvement of similar metabolites in plasma, urine and in NEC studies, and between NEC and IBD, suggests alterations to be associated with pathophysiological processes of intestinal and/or systemic inflammation^15,22,26,28^. Studies should be designed to explore the longitudinal evolution of AAA towards clinical onset of NEC, discriminating LOS-associated from non-LOS-associated NEC, as well as from NEC-free LOS. Samples should not only be collected in the first month of life, but until ca. 6 weeks of postnatal age, in order to include infants developing NEC beyond the first 4 weeks. The currently identified limited number of discriminating AAA should be validated in combination with existing clinical and biochemical risk assessment tools. This could be explored in different biospecimens, with a preference for urine and feces over plasma or serum, due to the non-invasive character of sampling. In parallel, fundamental research with simultaneous collection of feces, serum and urine samples could provide the possibility to study the kinetics of changes in the different compartments.

## Conclusion

In this case–control study, seven fecal AAA, of which six essential amino acids, were altered in the 3 days before clinical onset of severe NEC in preterm infants. AAA profiles were moderately accurate in the prediction of NEC. Further research should point towards validation of the role in the NEC diagnostic work-up, preferably in combination with existing risk index tools, and also taking NEC II stages into account.

## Methods

### Subjects

This study was embedded in an ongoing prospective multicenter cohort study in infants born before 30 weeks of gestation in nine participating neonatal intensive care units in the Netherlands and Belgium, with the primary objective of identifying novel non-invasive biomarkers for LOS and NEC^42,43^. Of all included infants, clinical data and fecal samples were collected daily from birth up to 28 days postnatally.

For the current case–control study, infants of the Dutch cohort born between February 1st, 2013 and July 31st, 2020 with confirmed severe NEC (modified Bell’s stage ≥ IIIA), and an equal number of control infants, were included. All infants with NEC suspected by the treating clinician, were retrospectively reviewed and staged by two expert clinicians (HN and TM) based on clinical, radiographic, biochemical, and if applicable, pathological data, conform the modified Bell’s criteria by Kliegman and Walsch^34^. The date of onset was defined as the day NEC was diagnosed, based on clinical and radiographic symptoms (e.g. intestinal pneumatosis). The stage was defined as the most severe stage within the NEC episode. In case of discrepancy, cases were re-evaluated until consensus was reached.

Infants were excluded in case of major congenital gastrointestinal diseases (e.g. anal atresia, duodenal atresia, Hirschsprung’s disease), LOS in the control group (as defined by the *Vermont* *Oxford* *Network),* NEC modified Bell’s stage I and II, abdominal surgery unrelated to NEC, or in case no fecal samples were available^44^. The study was approved by all local Medical Ethical Review Boards (protocol number A2016.313) from participating centers. Written informed consent was obtained from the parents or legal caretakers of included infants. Research was performed according to European and local guidelines and regulations, and in accordance with the Declaration of Helsinki.

### Study groups

Every NEC IIIA and IIIB case was matched to one control patient who had no symptoms of NEC, nor LOS prior to fecal sampling. Infants were matched based on gestational age (± 4 days) and samples were matched on postnatal age (± 1 day).

### Sample and data collection

Fecal samples were collected daily from the infant’s diaper by nursing staff members. The feces was transferred in a container (Stuhlgefäß 10 mL, Frickenhausen, Germany) and subsequently stored at − 20 °C within one hour after collection, until further handling. In case of multiple stool productions per day, the first fecal sample was stored. Sample collection was prematurely ceased in case of transfer to a referral hospital or demise before the postnatal age of 28 days.

In addition, demographic and detailed clinical data were collected daily, including administration of antibiotics, co-morbidities, mechanical ventilation and feeding practice. Feeding practice was assessed from birth to time of sampling. Based on the average daily percentage of human milk (HM) and formula milk (FM) from birth to sampling, feeding practice was classified as follows: (1) HM (including own mother’s and donor milk) (> 75% of the daily intake), (2) FM (> 75% of daily intake), and (3) a combination of HM and FM. In none of the participating centers, probiotics were administered routinely.

### Sample preparation and metabolic analysis by targeted high-performance liquid chromatography

Stored stool samples produced three (t_-3_), two (t_-2_) and one (t_-1_) day(s) prior to clinical onset (t_0_) of NEC IIIA and IIIB cases, and gestational and postnatal age-matched control samples, were selected for further analysis by targeted HPLC.

Approximately 150 mg feces was mixed with H_2_O/MeOH (1/1, v/v) in a 150 mg/500 µL ratio and was placed in a mixture machine for 10 min (IKA Vibrax VXR basic, Staufen, Germany) (modified from Erben et al. 2021)^45^. Subsequently, the samples were placed in an ultrasonic bath for 15 min. Then, the samples underwent two freeze–thaw-cycles by storing the samples in a − 80 °C freezer for 30 min and subsequently thawing at room temperature for 30 min. After the second cycle, the samples were homogenized by vortex and then centrifuged for 5 min at 2000 rpm. A volume of 500 µL of the supernatant was then transferred to a microcentrifuge tube for analyses. The supernatant was mixed with an internal standard solution in a 1:1 ratio. Finally, this mixture was centrifuged for 5 min at 10,000×*g* (6 °C) and filtered (Whatman, Buckinghamshire, UK) into compatible containers for the final analyses (Biochrom 30, Biochrome, Cambridge, United Kingdom)^45^.

Compounds were separated by ion-exchange chromatography, detected by UV absorbance after postcolumn derivatization with ninhydrin and analysed by means of targeted HPLC^46^. This technique allows for identification of 42 metabolites (Supplemental Table S1).

### Statistical analyses

#### Clinical and demographical data

For the statistical analyses of clinical and demographical data, Statistical Packages for Social Science (SPSS) (version 26.0, IBM, NY) was used. The distribution of the data was assessed by the shape of the histogram and in case of uncertainty, Shapiro Wilk test was applied. Parametric continuous data were presented as mean and standard deviation (SD), and were analyzed by independent samples Student’s t-test. Non-parametric continuous data were displayed as median and interquartile range (IQR) and analyzed by Mann–Whitney U test. Categorical data were reported as numbers and percentages and tested by chi-squared test or Fisher’s exact test.

#### Fecal amino acid and amino alcohol analysis

The statistical analyses of AAA profiles in relation to NEC were conducted using R version 4.0.2, packages: glmnet (v4.0-2) and pROC (v1.16.2).

Only compounds quantifiable in at least 50% of the fecal samples at the three predefined time points, in at least one of both study groups, were included for further statistical analysis^26^. Largely unquantifiable compounds (< 5 µmol/L) were excluded because they would have a low power, while increasing multiple testing burden.

Before further analysis, all metabolite measurements were calculated relatively to the total AAA concentration. Then, to avoid effect of potential drifting of the measuring device between the measurement performed in 2019 and 2020, a correction for both batches was performed by calculating the mean relative concentration per metabolite per batch and subtract this from the relative concentration per sample. On these corrected AAA data both a univariate association analysis and multivariate predictive analysis was performed.

##### Univariate association analysis

For the univariate analysis, a standard likelihood ratio test in a logistic regression model was performed after simplifying the data set by calculating the mean corrected AAA across time per study subject. We tested whether the corrected AAA values fit the regression model, with 0/1 response coded as control/case, significantly better than a null-model. The raw p value for each included amino acid was corrected for multiple testing by Benjamini–Hochberg false discovery rate (BH-FDR). BH-FDR adjusted *p* value < 0.1 was considered statistically significant.

##### Multivariate predictive analysis

The applied multivariate predictive model is a weighted logistic lasso regression. All non-missing relative measurements were used. Because not every included child passed stools on daily bases during the study period, there was a different number of analyzed fecal samples per child. As samples within a participant are likely dependent, and hence the amount of information per participant is not proportional to the number of samples per participant, samples were weighted. Input data were weighted based on the number of measurements per individual: if only one sample was analyzed from a given participant, the weight in the lasso regression analysis was 1. If two samples were available, the weight per sample was √(1⁄2), and for 3 samples per child, √(1⁄3) per sample. This renders the total weight of a participant with 1, 2, 3 samples equal to 1, 2*√(1⁄2) = √2, 3*√(1⁄3) = √3, respectively^47^. After batch correction, as described before, amino metabolite results were scaled by z-score. To avoid optimism bias, all measurements corresponding to one individual are simultaneously left out. Leave-one-out cross-validation (LOOCV) was used to evaluate predictive performance by area-under-the-ROC-curve (AUC). We tested whether the model classified significantly better than random by applying the exact same procedure to permuted labels (1000 permutations), and comparing the observed AUC to the distribution of random AUCs.

#### Sample size calculation

To our knowledge, there are no previous studies exploring targeted AAA profiles in feces in a preterm population with NEC. A formal sample size calculation could not be performed due to lack of previous data on this topic.

### Ethical approval

The study protocol was reviewed and approved by the local institutional review board of Amsterdam UMC, location VUmc, Amsterdam, the Netherlands (approval number A2020.190). 

### Informed consent

Written informed consent was obtained from both parents and/or legal guardians of all infants.

## Supplementary Information

Supplementary Information 1.

Supplementary Information 2.

## Data Availability

The data used in the current study are not publicly available but are available upon reasonable request.
