# Supplementary material for: Fecal amine metabolite analysis before onset of severe necrotizing enterocolitis in preterm infants: a prospective case–control study
Source: Sci Rep. 2022 Jul 19;12:12310. doi: 10.1038/s41598-022-16351-8 (PMC9296556; doi:10.1038/s41598-022-16351-8)
Supplement: Supplementary file 1 — Supplementary Information 1. [file 41598_2022_16351_MOESM1_ESM.docx]

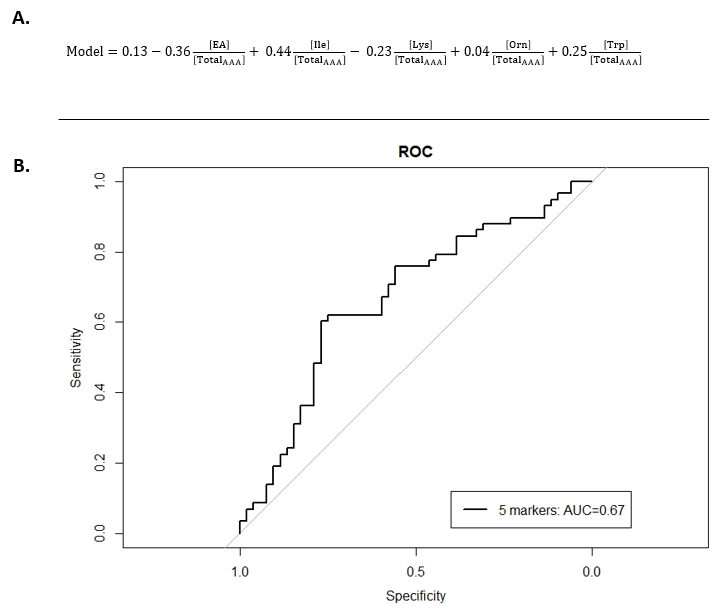


**Figure S1** Multivariate prediction analysis for necrotizing enterocolitis based on 5 amino metabolites. A. Rendered multivariate model based on ethanolamine, isoleucine, lysine, ornithine and tryptophan. B. Receiver operator characteristic (ROC) curve of the abovementioned model showing an accuracy (area under the curve, AUC) of 0.67 (p<0.001).

*AUC, area under the curve; EA, ethanolamine; Ile, isoleucine; Lys, lysine; Orn, ornithine; Trp, tryptophane*
